# Proximate Analysis of Poultry-Mix Formed Feed Using Maize Bran as a Base

**DOI:** 10.1155/2021/8894567

**Published:** 2021-09-21

**Authors:** Solagbade Saheed Afolabi, John Oluwafemi Oyeyode, Wasswa Shafik, Zubair. A. Sunusi, Adegoke Abdullahi Adeyemi

**Affiliations:** ^1^Department of Chemistry, Yazd University, P.O. Box 89175-741, Yazd, Iran; ^2^Polymer Department, Hussaini Adamu Federal Polytechnic, Kazaure, Jigawa, Nigeria; ^3^Department of Computing & Mathematics, King Fahd University of Petroleum & Minerals, Dhahran 31261, Saudi Arabia; ^4^Federal Government Girls' College, Kazaure, Jigawa, Nigeria; ^5^Biological Sciences Department, Federal University of Agriculture Abeokuta, Abeokuta, Nigeria

## Abstract

The purpose of this research was to demonstrate the proximate analysis of poultry-mix made using maize bran as a basis. Red beans, soya beans, and benny beans were the three samples utilised in this study. This work investigates the appropriate poultry mix for birds breed for meat and egg. Thirty grammes of proteinous feedstock were weighed and homogeneously combined with 70 grammes of maize bran. The following was revealed in a proximate analysis of the feeds: moisture ranged from 1.18% to 1.54%, unrefined lipids 0.99–3.08%, total carbohydrate 57% to 72%, ash content 38.48% to 38.92%, unrefined protein 18.38% to 22.53% and unrefined fiber 2.0% to 4.65% respectively for broilers and layers. In terms of nutritional concentrations, all feed samples showed a substantial variation. Based on the findings of the study, it can be stated that Soya bean-maize bran is an excellent poultry-mix formulation that has deep well-disposed benefits and meets nearly all nutritional needs for meat and egg-producing birds.

## 1. Introduction

For many years, the continual rise in the human population has had a significant impact on the demand for animal-based foods. In third-world nations like Nigeria, population growth has not kept pace with agricultural expansion, particularly in livestock output. Feedstocks like maize bran and soya bean are generally of high quality, providing all of the necessary nutrients in the right quantities for the poultry's healthy growth and development. Rice milling waste in broiler chicken diets significantly increased nutrient utilization and economic value, and may thus be utilised to replace maize in broiler chicken diets between 10% and 40% as demonstrated [1]. The world's livestock producers are having difficulties in meeting the demand for animal production, and this disparity has led to severe malnutrition [2].

It was reported years after that the number of undernourished people is still increasing world-wide by about five million each year with overwhelming majority in the developing world [3]. Unfortunately, the egg, which is a compact source of vital nutrients and should have been a more natural way of delivering the needed nutrients, is prohibitively expensive for the typical man. In the retail market, eggs had varying quality and nutritional content, which may impact human RDA fulfilment and perhaps enhance egg quality and nutritional value [4]. Numerous studies have been carried out for the proximate analysis of poultry-mix formed. To show this, the authors of [5] studied and evaluated the intake of low-graded-cassava meal, finding that the newly created drink “dusa” contained more protein than maize grains. Furthermore, an experiment was conducted to compare the energy sources of Cashew nut meal and conventional-plant-proteins meal. Based on the results of this investigation, Cashew nut in diets can be an excellent alternative to conventional-plant-proteins. [6].

To attain the exact quantities of nutrients, it is vital to balance the ratio of diets. The research's findings revealed substantial differences in the quality of chicken diets made from soya beans, red beans, and benny beans. It can be stated that Soya bean-maize bran proven to be an appropriate poultry-mix formulation with minimal side effects that meets nearly all nutritional needs for poultry birds. It has a high percentage of carbohydrates, which are an essential source of energy for broilers and layers. It also has a high proportion of natural protein, which is the fundamental ingredient of structural and protective tissues for poultry birds.

The main contribution of this paper is to derive a balanced diet that resolve to provide appropriate quantities of biologically available nutrients required by birds breed for meat and egg. Moisture content, ash content, unrefined protein, unrefined fiber content, and unrefined lipid content are all subjected to an experimental analysis. The total carbohydrate content of maize bran, red and soya beans, and the Benny, on the other hand, was determined in order to provide farmers with useful information on the nutritional composition of poultry feeds.

## 2. Materials and Methods

In this section, the used methodology and content samples are briefly laid that includes Sample Assortment and Preparation.

### 2.1. Sample Assortment and Preparation

The maize bran, red beans *(Phaseolus vulgaris)*, soya beans *(Glycine max),* and Benny seed *(Sesame indicum)* were bought from the local market in Masaka, Nassarawa state Nigeria.

### 2.2. Preparation of Poultry-Mix

A 30 g each of the proteinous feedstock was weighed and mixed homogeneously with 70 g of the maize bran, each detailed below:

### 2.3. Analysis for Moisture Content

A 5 g sample of poultry-mix was put into a crucible and placed in an oven at 105°C for 12 hours. After cooling in desiccator, the sample was re-weighed using the technique described by [7]. The percentage of moisture content (%) was calculated using,

(1) $$\begin{array}{c} 100\frac{\left(\beta -\alpha \right)-\left(\gamma -\alpha \right)}{\left(\beta -\alpha \right)}, \end{array}$$

where *α* is the weight of clean, dry crucible (g), *ß* is the weight of crucible + wet sample (g), *γ* is the weight of crucible + dry sample (g).

### 2.4. Analysis for Ash Content

A 5 g of the previously calcined sample obtained by heating at high temperature in the absent of air was weighed into a dry crucible and brought to constant weight. The crucible was placed in a furnace and heated for 12 hours at 550°C and was left to cool in a desiccator, after which the crucible with the ash was carefully weighed. The percentage of ash content was calculated using,

(2) $$\begin{array}{c} \text{Ash content}\left(\%\right)=100\frac{\alpha -\beta }{\gamma }, \end{array}$$

where *α* is the weight of crucible with sample (g), *ß* is the weight of crucible with ash (g), *γ* is the weight of sample (g).

### 2.5. Analysis for Lipid Content

Different types of lipids in nature differ structurally from one another. The qualitative analysis of the lipid was carried out as follow; The extraction flasks were removed from the kiln, it was exposed to cool by the use of a dryer, and lastly, it was weighed to milligrams. A 5 g of the dry sample was weighed into insertion thimble, with focal handling of the dried sample through tongs, and then the sample was positioned within the extraction unit. The flask that contained the hexane at 75% of totality volumes to an extractor was connected the solution brought to boiling and the heat was adjusted to attain almost ten refluxes within sixty minutes based on the lipids within the sample.

When all the above steps were done to that point, the hexane was meant to be evaporated in rotary-evaporator. The used flasks within the experiment were cooled in a desiccator, and the weighed respectively to within milligrams. The defatted samples were used in determining unrefined fiber. The Unrefined lipid was calculated using,

(3) $$\begin{array}{c} \text{Unrefined lipid content }\left(\%\right)=100\frac{\beta -\alpha }{\gamma }. \end{array}$$

Given that, *α* depict the weight of clean, dry flask (g), *ß* is the weight of flask with fat (g), *γ* is the weight of the sample (g).

### 2.6. Analysis for Fiber Content

Fiber content is the most important factor in determining how fiber composites behave. Acid hydrolysis was used to determine the crude fiber content. A 3 g defatted sample was carefully weighed and was placed in the 200 ml flask, then boiling sulphuric acid solution was added. The boiling point of the attached condenser was maintained, the sample was boiled for an accurately half an hour, the quantity of distilled water was kept steady and the flask was swirling from often in order to eliminate particles that may then attached to the sides. The filtration funnel was lined with the filter paper and pre-heated with boiling water. Finally, the flask was detached and allowed to rest for 60 seconds, then contents were filtered with suction.

Residual product from the treated filter paper was transferred into sodium hydroxide solution boiled for half hour. The hydrolyzed mixture from the crucible was carefully separated after standing for 1 min. The residue was washed with boiling water, with the HCI solution and then again with boiling water, finishing with three washes with hexane. The crucible was placed in a kiln set at 105°C for 12 hours then cooled in desiccator. The crucible was weighed quickly with the residue inside and was placed in the crucible furnace at 550°C for 3 hours and was left to cool in a desiccator and weighed again. Unrefined fiber was calculated using,

(4) $$\begin{array}{c} \text{Unrefined fiber content }\left(\%\right)=100\frac{\alpha -\beta }{\gamma } \end{array}$$

where, *α* is weight of crucible with dry residue (g), *ß* is the weight of crucible with ash (g), *γ* is the weight of sample (g).

### 2.7. Analysis for Protein Content

A 1 g of sample was weighed and transferred to a Kjeldahl flask; 10 g Na_2_SO_4_, 0.7 g HgO, 25 ml sulphuric acid and a few grains of pumice stone was added after which the flask was heated at 320°C, shaking irregularly until the material was carbonized and the bubbles disappeared, the temperature was raised to 360°C, then brought to a moderate bubble. The flask walls were not allowed to burn in order to prevent organic particles from adhering to them.

As a clear solution was observed with no color possessions, it was then boiled for 2 hours longer and cool. The distilled water of 350 ml was carefully added onto the flask, where the contents were stirred, given that some zinc pellets were added and left to cool. Considerably, the sulphuric acid with 0.5 N of the capacity of 25 ml was collected into a single flask of the distilling apparatus, anticipated nitrogen, with noticeable droppings of methyl red indicator added concurrently.

The experiment added both a 100 ml of the sodium hydroxide and 10 ml sodium sulphate to the sample in order to maintain ammonia where the solution is well mixed connected to the distilling apparatus. The flask was heated in order to distill approximately 150 ml of the liquids in half an hour. The use of litmus paper depicted alkaline, although the distillation process continues. The content in the flask was stirred irregularly. In the collecting flask, the excess sulphuric acid was titrated with sodium hydroxide 0.25 N, the methyl-red-indicator.

(5) $$\begin{array}{c} \text{Nitrogen in sample}\left(\%\right)=100\left[\frac{\alpha \times \beta }{\gamma }0.014\right], \end{array}$$

where, *α* is the chlorohydric acid used in titration (mL), *β* is the normality standard, *γ* is the weight of the sample.

### 2.8. Analysis for Carbohydrate Content (Clegg-Anthrone Method)

The 2.5 grams of the wet samples were ranged from 0.001 g to 300 mg of sum carbohydrates, transferred to a stopped of 100 ml graduated cylinder, with water of 10 mL capacity was added and stirred in combination glass rod to diffuse the samples. The solution was transferred to 1 mL spectrophotometer cells. The green color was found stable for a period of only 2 hours. The absorption-rates were read at 630 nm adjacent to the blank solution calculated [8].

(6) $$\begin{array}{c} \text{Total available carbohydrates}\left(\%\text{ of }{\text{C}}_{6}{\text{H}}_{12}{\text{O}}_{6}\right)=\frac{25.\beta }{\alpha .W}, \end{array}$$

Given that, W is the weight of the sample (g), *α* is the absorption of diluted standard, *ß* is the concentration of the sample.

## 3. Results and Discussion

The experimental results and discussion of the nutritional value of a poultry feed that are considered when formulating diets are unrefined protein, moisture, unrefined lipid, unrefined fiber, ash and carbohydrate content.

### 3.1. Unrefined Lipid Content of Poultry-Mix

**Figure 1:**
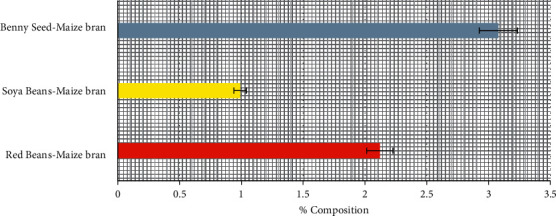
Percentage unrefined lipid content of poultry-mix.

The result in Figure 1 indicates that Soya beans-maize bran mix has the lowest unrefined lipid content of 0.99% while the Benny seed-maize bran and red beans-maize bran has an unrefined lipid content of 3.08% and 2.12% respectively. Poultry-mix with high unrefined lipid content will be ideal for poultry birds raised for meat; hence Benny seed and red bean-maize bran will be a good choice for such poultry farmer/farm. In poultry farms where fat is not a superior demand, soya bean-maize bran will be appropriate as it will provide the required energy needed by the birds [9].

### 3.2. Moisture Content of Poultry-Mix

**Figure 2:**
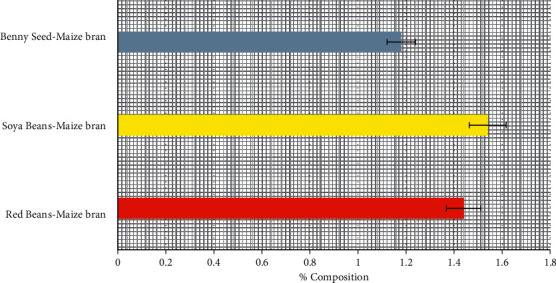
Percentage moisture content of poultry-mix.

The result in Figure 2 indicates that Soya bean-maize bran and red bean-maize bran mix have a high moisture content of 1.54% and 1.44% respectively compared to Benny seed-maize bran, which has a moisture content of 1.18%. Moisture content affects the physical and chemical aspects of the feed, which relates its freshness and stability for the storage of the feed over a long period of time [10]. Therefore, it is advisable to formulate feed with low moisture content for feeding poultry birds to keep deterioration in the feed to a minimum during storage.

### 3.3. Total Carbohydrate Content of Poultry-Mix

**Figure 3:**
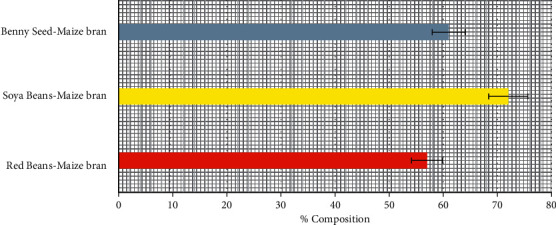
Percentage total carbohydrate content of poultry-mix.

The Soya bean-maize bran and Benny seed-maize bran contained a high amount of carbohydrate with 72% and 61% respectively, while, the red bean-maize bran contained 57%, as shown in Figure 3. This research showed that Soya bean-maize bran and Benny seed-maize bran are good sources of carbohydrate in poultry diet formulation considering their percentages, which are 72% and 61%, respectively. Using Red bean-maize bran, the level of carbohydrate would be low in the feed, which would not be beneficial to broilers and layers in terms of health issues [11].

### 3.4. Ash Content of Poultry-Mix

**Figure 4:**
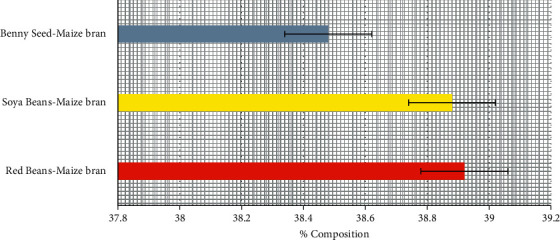
Percentage ash content of poultry-mix.

The result for the ash content in Figure 4 showed that Red bean-maize bran, Soya bean-maize bran, and Benny seed-maize bran, had ash content to be 38.92%, 38.88% and 38.48% respectively. The three samples had an approximate amount of ash content to be 39.0%. Too much ash in a poultry diet may cause crystals that are being formed within the urinary-tracts, excluding the kidneys, and the bladder, specifically in poultry faunas that ensure kidney diseases, also excess ash content causes the bone and joint problems in growing poultry birds. The poultry industry's future growth is threatened by several factors, including immunity, health, and production. The existing state of the business, as well as the development and re-emergence of illnesses, will continue to be important issues as depict [12].

Nevertheless, the feeds with stumpy ash-content are co-operative in monitoring the urinary-tract complications, literature have not recorded that feeds that are high in ash-content are beneficial to poultry birds that are being raised for meat and egg [12]. Therefore, all three samples are good choices for poultry-mix formulation considering their ash content but different mineral compositions.

### 3.5. Unrefined Protein Content of Poultry-Mix

**Figure 5:**
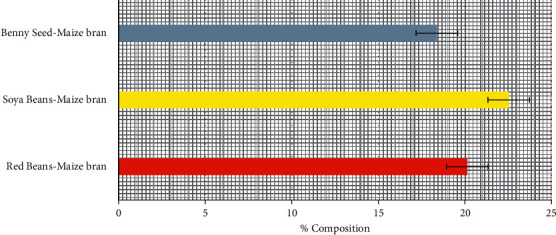
Percentage unrefined protein content of poultry-mix.

Soya bean-maize bran had a slightly high amount of unrefined protein to be 22.53% compared to Red bean-maize bran and Benny seed-maize bran with 20.13% and 18.38% respectively as shown in Figure 5. A high amount of unrefined protein contains the main crucial components of essential, and protecting soft tissue, like the skin feather, the bone matrix, and the ligaments and the soft tissues comprising structures and muscle for both broilers and layers, where animal addittives were put under consideration [13].

Higher altitudes of unrefined protein advance effectiveness by accumulative broiler enactment, and the dispensation yield. Nonetheless, when protein is inadequate in poultry feed as seen in the case of using Benny seed-maize bran, there is a decrease of growth or even efficiency and an insertion of protein from a smaller amount of vigorous body tissues to preserve the utilities of additional vigorous tissues. In chicken feed, crude protein is an important nutrient. Lowering the usage of crude protein not only lowers feed costs, but it also reduces pollution in the chicken industry as illustrated [14]. Given its protein content (22.53%), soya bean-maize bran is an excellent source of protein in poultry diets.

### 3.6. Unrefined Fiber Content of Poultry-Mix

**Figure 6:**
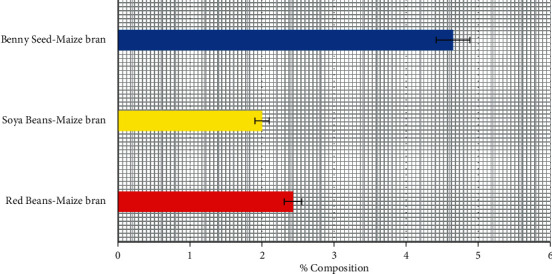
Percentage of unrefined fiber content of poultry-mix.

Benny seed-maize bran contained the highest amount of unrefined fiber to be 4.65%, Soya beans-maize bran, and red bean-maize bran contained a low amount of unrefined fiber to be 2.0% and 2.43% respectively as shown in Figure 6. Poultry feeds high in unrefined fiber should be discouraged due to the adverse effects on nutrient, utilization, and performance such as a decrease in body weight gain and feed conversion [14]. Therefore, any poultry farmer that is breeding poultry birds raised for meat and egg should ensure that the poultry-mix should be low in unrefined fiber and these shows why Soya bean-maize bran is an ideal poultry feed considering its low unrefined fiber content (2.0%) as demonstration [15].

## 4. Conclusion

The relatively low percentage moisture content indicates low susceptibility of micro-organisms and with a low probability of affecting the physical and chemical aspects of the feed, which relates its freshness and stability for the storage of the feed over a long period of time. Furthermore, it has a low percentage of unrefined lipids, which signifies that where fat is not a significant demand, it would be appropriate to provide the required energy needed by the birds. The diets for broilers and layers at the same, but the layers diet typically has slightly more calcium and is fortified with extra vitamins for proper embryo development and egg-shell formation. In contrast, broilers require high protein, calcium, metabolizable energy and less fiber in their feed. Therefore, it can be concluded that soya bean-maize bran, which has all these requirements for layers and broiler feed, can be used. As our future research, this experiment is to be subjected to other chemical content analysis of poultry feeds mix in aspect to increase poultry productivity.

## Data Availability

All the data that was used to support the results of this study are encompassed within the paper.

## References

[1] Onabanjo R. S., Ojewola G. S., Onunkwo D. N. (2021). Performance of broiler chickens fed rice milling waste based diets. *Nigerian Journal of Animal Production*.

[2] Lokaewmanee K., Seedarak K. (2021). The effects of dietary maoberry (Antidesma sp.) pomace supplementation on broiler growth performance, blood parameters, carcass quality and intestinal histology. *Indian Journal of Animal Research*.

[3] Elgar F. J., Sen A., Gariépy G. (2021). Food insecurity, state fragility and youth mental health: a global perspective. *SSM-Population Health*.

[4] Hossain M., Rahman W., Ali M., Sultana T., Hossain K. (2021). Identification and antibiogram assay of *Escherichia coli* isolated from chicken eggs. *Journal of Bio-Science*.

[5] Kirrella A. A., Abdo S. E., El-Naggar K. (2021). Use of corn silk meal in broiler diet: effect on growth performance, blood biochemistry, immunological responses, and growth-related gene expression. *Animals*.

[6] Ocheja J. O., Halilu A., Shittu B. A., Eniolorunda S. E., Ajagbe A. D. (2021). Haematology and serum biochemistry of yearling west african dwarf goats fed cashew nut shell based diets. *Veterinary Medicine and Public Health Journal*.

[7] Różewicz M. (2019). Production, use and efficiency of utilising grains of various cereal species as feed resources for poultry production. *Polish Journal of Agronomy*.

[8] Krishnan V., Awana M., Singh A. (2021). Starch molecular configuration and starch-sugar homeostasis: key determinants of sweet sensory perception and starch hydrolysis in pearl millet (Pennisetum glaucum). *International Journal of Biological Macromolecules*.

[9] Ćirović A., Ivanovski I., Ćirović A., Nikolić D., Ivanovski A. (2021). The adjuvant aluminum fate-metabolic tale based on the basics of Chemistry and biochemistry. *Journal of Trace Elements in Medicine & Biology*.

[10] Kačániová M., Hleba L., Džugan M., Pasternakiewicz A., Kňazovická V. (2021). Microbiological properties and antimicrobial effect of Slovakian and Polish honey having regard to the water activity and water content. *Journal of Microbiology, Biotechnology and Food Sciences*.

[11] Hafez H. M., Attia Y. A. (2020). Challenges to the poultry industry: current perspectives and strategic future after the COVID-19 outbreak. *Frontiers in veterinary science*.

[12] Tobin G., Schuhmacher A. (2021). Laboratory animal nutrition in routine husbandry and experimental and regulatory studies. *Handbook of Laboratory Animal Science*.

[13] Man K. Y., Chow K. L., Man Y. B., Mo W. Y., Wong M. H. (2021). Use of biochar as feed supplements for animal farming. *Critical Reviews in Environmental Science and Technology*.

[14] Attia Y. A., Bovera F., Wang J., Al-Harthi M. A., Kim W. K. (2020). Multiple amino acid supplementations to low-protein diets: effect on performance, carcass yield, meat quality and nitrogen excretion of finishing broilers under hot climate conditions. *Animals*.

[15] Giri A., Ranjan P., Bharti V. K. (2021). Selenium toxicity in domestic animals: sources, toxicopathology, and control measure. *Selenium Contamination in Water*.

